# Mixed Epithelial and Stromal Tumor of the Kidney with Extension into Inferior Vena Cava: Case Report and Discussion of Adult Biphasic Cystic Renal Lesions and the Significance of Vascular Involvement

**DOI:** 10.1155/2018/8234295

**Published:** 2018-10-01

**Authors:** Maria M. Picken, Davide Bova, Michael R. Pins, Marcus L. Quek

**Affiliations:** ^1^Department of Pathology, Loyola University Medical Center, Chicago, USA; ^2^Department of Radiology, Loyola University Medical Center, Chicago, USA; ^3^Advocate Lutheran General Hospital, Department of Pathology, USA; ^4^Department of Urology, Loyola University Medical Center, Chicago, USA

## Abstract

Mixed epithelial and stromal tumor (MEST) is a biphasic adult renal lesion composed of solid and cystic areas containing spindle cell stroma and epithelium that lines the tubules and cystic spaces. While most MEST lesions are benign, rare cases with malignant morphology and biology have been reported. We present a case of mixed epithelial and stromal tumor of the kidney (MEST) with extension into the inferior vena cava in a young adult male. We discuss the differential diagnosis of MEST in the context of other biphasic cystic renal lesions and the significance of vascular involvement in the setting of an otherwise benign tumor morphology.

## 1. Introduction

Mixed epithelial and stromal tumor (MEST) is a biphasic adult renal lesion containing solid and cystic areas composed of spindle cell stroma and epithelium that lines the tubules and cystic spaces [1–9]. Currently, MEST is included in the “mixed epithelial and stromal tumor family” of tumors in adults, which comprises a spectrum of tumors ranging from predominantly cystic tumors (adult cystic nephromas) to tumors that are variably solid and cystic (MEST) [10]. Based on the molecular studies thus far published, MEST and adult cystic nephroma are similar to each other but different from other kidney lesions, including morphologically similar cystic and biphasic pediatric lesions [8, 11–14]. While most MEST lesions are benign, rare cases with malignant transformation have been reported [15–22]. In this report, we present a case of morphologically benign mixed epithelial and stromal tumor of the kidney (MEST) with inferior vena cava involvement in a young adult male.

## 2. Case Report

A 27-year-old male presented with a 1-day history of intermittent gross painless hematuria. His past medical history included herniated lumbosacral disk with radiculopathy, otherwise unremarkable. Social history included current smoking, 6 pack/year, and occasional EtOH. The patient was single and had no children; family history was negative for genitourinary malignancies. Physical examination was unremarkable with BMI 23 and BP 120/86 and no prescribed medicines or drug use. Laboratory tests showed normal CBC, normal coagulation profile, and normal renal function.

**Figure 1:**
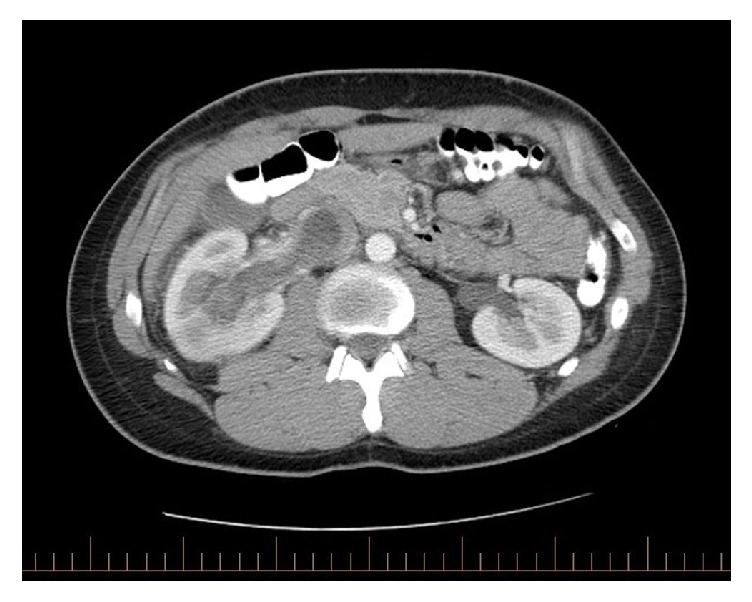
Axial, contrast-enhanced CT demonstrates a centrally located lobulated mass invading the renal vein and extending into the lumen of the inferior vena cava.

Axial, contrast-enhanced CT demonstrated a centrally located, 4 x 4 x 4.6 cm, lobulated mass invading the renal vein and extending into the lumen of the infrahepatic inferior vena cava (Figure 1). CT angiography of the chest showed no evidence of detectable pulmonary emboli and bone scan was negative for metastases.

Right radical nephrectomy, partial adrenalectomy, inferior vena cava tumor thrombectomy (infrahepatic), and extended retroperitoneal lymphadenectomy were performed; flexible cystoscopy performed during this surgery showed a bulbar urethral stricture (not clinically significant) and otherwise normal bladder. The intravascular tumor pedicle was easily removed intact from the vein lumen by pulling.

**Figure 2:**
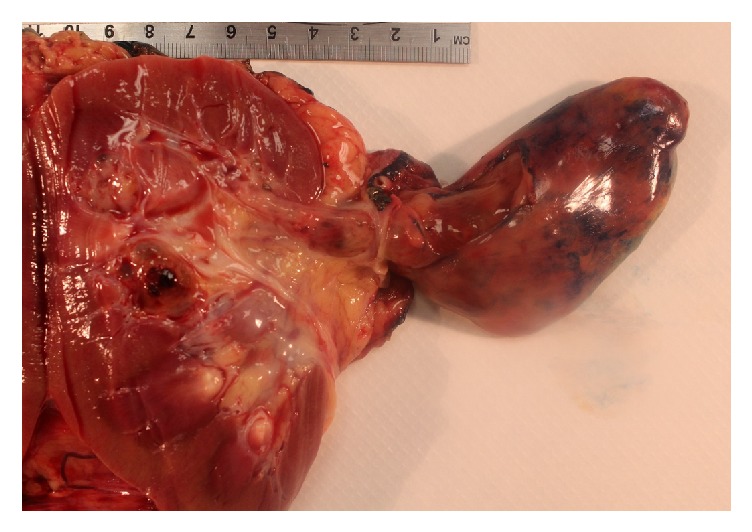
Gross image of the intrarenal mass and an elongated pedicle extending into the lumen of the inferior vena cava. The mass is lobulated and partially cystic and the pedicle has a smooth border.

Gross examination of the nephrectomy specimen demonstrated a centrally located tumor with no gross invasion of adjacent tissue but with the pedicle extending into the inferior vena cava (Figure 2). Grossly, the tumor pedicle had a smooth surface and no attachment to the renal vein. Grossly, the mass was partially cystic with variably sized cysts with a smooth lining. The intervening stroma formed grossly discernible nodules of variable thickness. No tumor necrosis was grossly apparent. The lesion appeared to be well demarcated with no invasion of adjacent kidney parenchyma.

**Figure 3:**
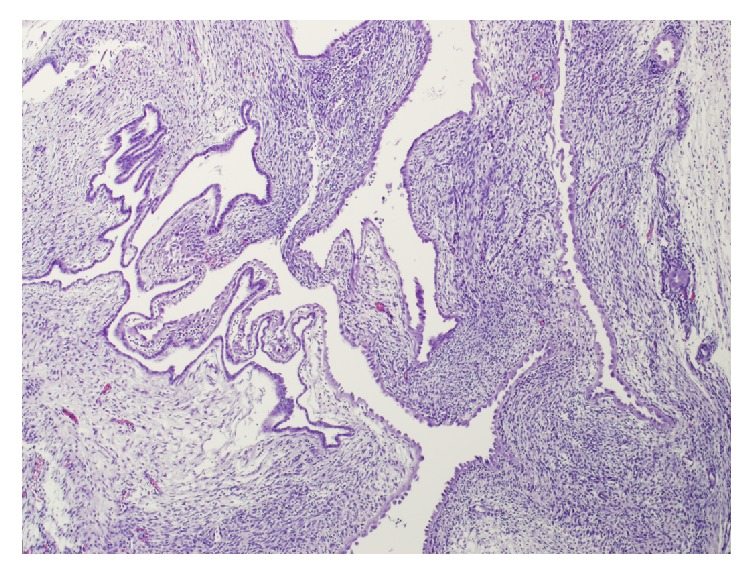
Cystic and biphasic tumor with benign, spindle cell stroma, focally resembling ovarian stroma, arranged with increased density in pericystic areas. Epithelial lining of cystic spaces is composed of a monolayer with cuboidal, flattened, and, focally, a hobnail appearance. No morphologic features of dysplasia are identified. Hematoxylin and eosin original magnification: 300x.

Microscopically, the tumor was well demarcated with an elongated pedicle bulging into the renal pelvis and renal vein and a biphasic morphology with spindle cell stroma and a benign epithelial monolayer lining the cystic spaces (Figure 3). The stromal component was composed of uniform spindle cells without cellular atypia, necrosis, or mitoses (Figure 3). Focally, the stroma was densely cellular, resembling ovarian stroma, but no areas of scarring or fibrosis resembling corpora albicantia of the ovary were identified (Figure 3). No blastemal, skeletal muscle or clusters of clear cells were seen. The cystic spaces were lined by a single layer of epithelium, which was cuboidal or flattened or, focally, had a hobnail appearance (Figure 3).

**Figure 4:**
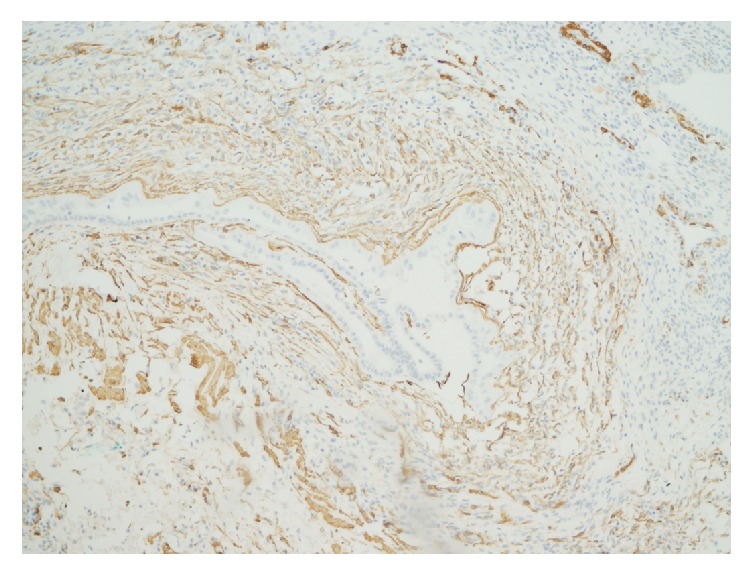
The spindle cell stroma diffusely positive for smooth muscle actin. Immunoperoxidase stain for smooth muscle actin; original magnification: 125x.

**Figure 5:**
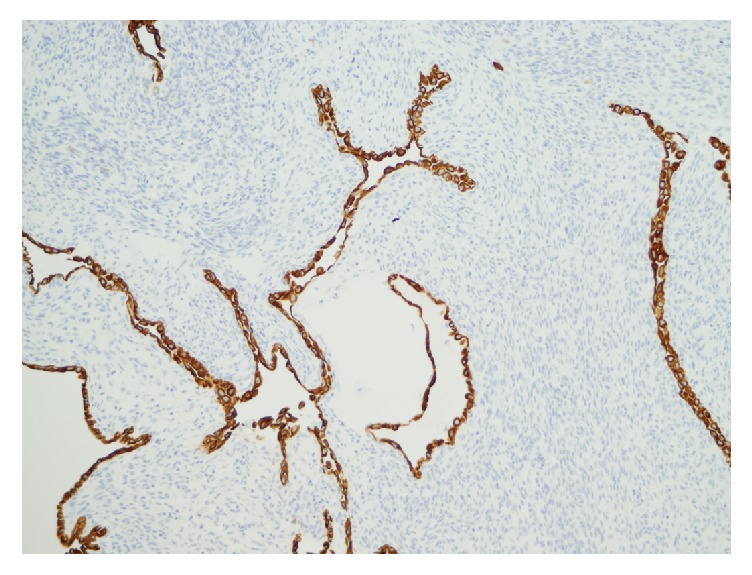
Epithelium lining of cystic spaces showing diffuse and strong positivity for CK7. Immnuoperoxidase stain for CK7; original magnification: 200x.

The stromal cells were diffusely and uniformly positive for SMA (Figure 4) and desmin and, focally, for CD10, while stains for inhibin, CD34, WT-1, S-100, MART1, and HMB-45 were negative. The epithelial component was positive for CK7 (Figure 5), for PAX-8, and, focally, for CD10. Immunostains for ER and PR were negative in stromal and epithelial components. The ki-67 index was low (<2%).

**Figure 6:**
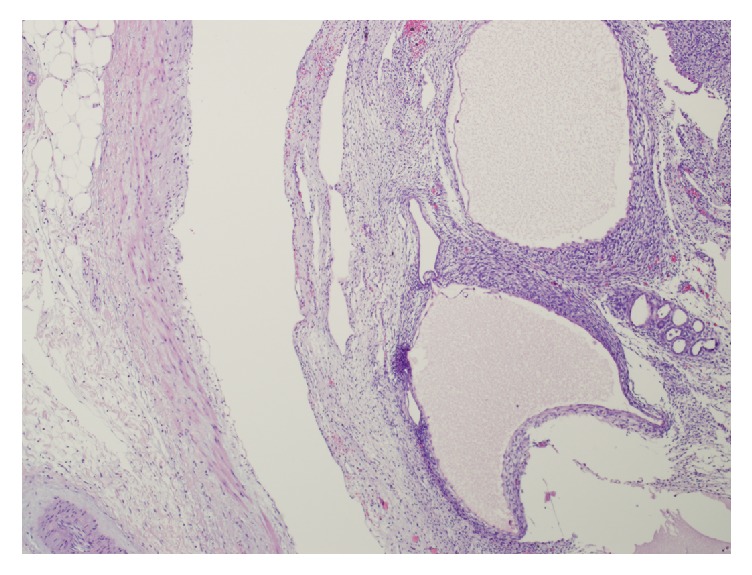
Tumor within the renal vein lumen with tumor pedicle seen on the right and vessel wall on the left. Hematoxylin and eosin original magnification: 300x.

The tumor pedicle extending into the inferior vena cava showed similar morphology except for some edema and a focal procedure-related hemorrhage. Specifically, no epithelioid morphology and no tumor necrosis or mitoses were seen despite extensive sampling. The pedicle appeared to be floating in the vascular lumen without attachment to, or invasion of, the vascular wall (Figure 6). The outer surface of the tumor pedicle was covered by endothelial (CD31/CD34 positive) cells (not shown).

FISH studies for* ETV6* rearrangement by an* ETV6* break-apart probe on chromosome 12 at 12p13.2 and for* SS18* by a synovial sarcoma break-apart probe on chromosome 18q11.2 were negative.

A diagnosis of “mixed epithelial and stromal tumor (MEST) of the kidney with extension into IVC” was rendered. After surgery, the patient recovered uneventfully and no recurrences have been reported at 3 years' follow-up.

## 3. Discussion

MEST is a rare, adult, biphasic tumor of the kidney [1–10]. While most cases behave benignly, very rare malignancies have been reported [15–22]. While the tumor described in this report showed many classic features typically seen in benign MEST, the visible tumor extension into the inferior vena cava was highly unusual.

MEST tumors are well circumscribed, range widely in size (from 2 cm to 24 cm), and typically show no gross invasion of adjacent tissue, which was also the case in the tumor described in this report. Typically for MEST, the current tumor was centrally located, with involvement of the renal pelvis, and displayed a cystic architecture with grossly apparent stromal nodules [1–10]. Microscopically, both stromal and epithelial components appeared benign. The stroma contained fibrous-appearing, leiomyoma-like, and/or ovarian-like areas [1–10]. The epithelium lining the cystic cavities and tubular spaces formed a monolayer ranging from hobnailed to cuboidal to flattened; on rare occasions, intestinal or cervical differentiation was also reported [23, 24].

As seen in MEST tumors, the stroma in the current tumor was also positive for smooth muscle markers (SMA and desmin), while the ovarian-like areas were positive for CD10 and melanocytic markers (HMB-45 and MART1 ) were negative [1–10]. The epithelial components were positive for PAX8, for CK7, and focally for CD10. While, in the majority of MEST tumors, positivity for ER/PR has been reported, in close to 40% of tumors these markers were negative [6] and the tumor described in this report was also negative.

Clinically, most MEST tumors have been reported in perimenopausal women (mean age of 52 years) but rare male patients and exceptionally rare cases of older children have also been reported [25–27]. Similarly, long-term hormonal treatment/exposure, suspected of involvement in tumor development, has been frequently but not invariably identified. Our patient was male, with no history of obesity or hormonal exposure. However, the possibility of a dietary intake of hormones (meat of animals raised on hormones) or exposure to plastics should be considered and it has been proposed as a mechanism in men with no history of medical hormonal exposure [6, 21, 25].

While most MEST are benign, rare malignant tumors (17 cases thus far) have been reported [15–22, 28]. Thus, in the current case, in view of the renal vein involvement, both benign and malignant biphasic tumors were considered in the differential diagnosis.

Angiomyolipoma with epithelial cysts (AMLEC) is a smooth-muscle-predominant (or “fat-poor”) angiomyolipoma, which also contains epithelial cysts and displays mixed, solid, and cystic architecture [29, 30]. Thus, both lesions, AMLEC and MEST, are morphologically similar. However, positivity for melanocytic markers in AMLEC and negativity in MEST distinguishes these lesions and these markers were also negative in the tumor of this report.

A “**S**mooth  **M**uscle and  **A**denoma-like  **R**enal  **T**umor” (SMART), comprised of smooth muscle stroma and complex but cytologically benign, epithelium-forming, tubulopapillary, adenoma-like nodules, rather than the monolayer typically seen MEST, has recently been described [31]. While the authors consider SMART a distinct entity, given the phenotypic overlap, it may be considered a variant of MEST. To this end, we reported, previously, a rare MEST malignancy associated with focal papillary renal cell carcinoma arising in one of the cysts [21]. In the case under discussion, however, epithelium lining the cysts formed a monolayer. Overall, however, a carcinomatous component in MEST was only reported thus far in 3 out of 17 MEST malignancies [21, 28].

Congenital mesoblastic nephroma (CMN), the most common congenital renal neoplasm, also shows a biphasic architecture with cysts and tubules embedded in abundant spindle cell stroma and, hence, is morphologically very similar to MEST. In fact, earlier literature reports of “adult mesoblastic nephroma tumors” most probably represent MEST. Thus, currently, according to the 2016 WHO classification of renal tumors, the distinction between these two entities is based on the patient's age, with CMN being considered a distinctly pediatric tumor [10]. CMN can be of classic, cellular, or mixed types. While the classic type is typically diploid and has an excellent prognosis, the cellular type may show aneuploidy and may recur.

CMN of the cellular type has a specific chromosomal translocation, which leads to fusion of the* ETV6-NTRK3* genes. The latter has not been detected in MEST thus far studied and, in our tumor, FISH was negative for ETV6 rearrangement [10, 32].

Among MEST malignancies, 14 out of 17 reported tumors showed stromal malignancy with features of undifferentiated sarcoma, which were synovial sarcoma-like or, on rare occasions, possessed rhabdomyosarcoma-like and chondrosarcoma-like features [15–21, 28]. In one tumor, sarcomatous change was present only focally in about 20% of the sectioned tumor, while most of the cells in the stroma were bland and appeared benign [16]. In the case under discussion, no malignant features were seen, despite extensive sampling.

Malignant MEST with a sarcomatous stromal component shows morphologic overlap with primary renal synovial sarcoma (RSS) (formerly “embryonal sarcoma of the kidney”) [33]. While most RSSs are monophasic, these tumors can entrap native renal tubules, some of which may become cystically dilated. Most RSS carry the* SS18-SSX2* gene fusion which has not, thus far, been detected in malignant MEST tumors thus far studied [10, 17, 32]. In our patient, FISH studies for* SS18* rearrangement using a synovial sarcoma break-apart probe on chromosome 18q11.2 were negative.

Thus far, a diagnosis of MEST appears to be based on the exclusion of other tumors with overlapping morphologies, both benign and malignant. Also, no MEST-specific molecular signatures have been detected and, among the discriminating factors in the differential diagnosis, the patient's age appears to play a seemingly decisive role [10–14]. While this is rather convenient, it lacks specificity/precision.

Among the poor prognostic factors in renal tumors, advanced stage, margin status, and renal sinus/vascular involvement are routinely considered. The current case showed a morphologically benign MEST with tumor extension into the IVC. However, in view of the apparently benign tumor morphology, we interpreted intravascular extension as an unusual tumor growth pattern rather than an indication of malignancy.

The intravascular component of the tumor showed no features of malignancy: no epithelioid features in the smooth muscle stroma, no necrosis, and no features of sarcoma; the only notable feature of the tumor was hemorrhage, both old and recent. Two other cases of morphologically benign MEST with renal vein involvement were also recently reported and both were apparently also clinically benign [32, 34]. Interestingly, on rare occasions, MEST has been noted to form a long pedicle, extending not only into the renal pelvis but even into the ureter [35].

A similar situation, with a seemingly benign tumor involving the renal vein, has been reported in rare cases of renal oncocytoma. Renal oncocytomas with intravascular extension into the renal vein [36] did not show morphologic, immunohistochemical, or cytogenetic differences from their counterparts without evidence of intravascular invasion. However, caution is advised, since, recently, a case of a renal oncocytoma with vascular extension and liver metastases was reported [37]. While, in our case, the follow-up period is relatively short (<3 years), the absence of metastases suggests an overall benign behavior of this tumor. However, the clinical significance of such tumor extension is at present unknown and a longer follow-up may be needed to determine the biology of such tumors. Caution is advised since MEST tumors have been reported to recur locally after incomplete excision [38] and peritoneal seeding following incomplete resection, resulting in a separate paracolonic MEST, has also been reported [39].

## 4. Conclusions

A case of MEST with inferior vena cava involvement lacking cytologic features of malignancy appeared to be a benign tumor. However, caution is advised in the management of MEST, since incomplete tumor resection can lead to recurrence and, in rare cases, malignant transformation can occur with a grim prognosis. Hence, a careful long-term follow-up is warranted. Moreover, caution is advised in the management of patients based on limited tumor sampling, such as core biopsy or cytology.
